# Targeted deletion of Atg5 in chondrocytes promotes age-related osteoarthritis

**DOI:** 10.1136/annrheumdis-2015-207742

**Published:** 2015-10-05

**Authors:** Thibault Bouderlique, Karuna K Vuppalapati, Phillip T Newton, Lei Li, Björn Barenius, Andrei S Chagin

**Affiliations:** 1 Department of Physiology and Pharmacology, Karolinska Institutet, Stockholm, Sweden; 2 Department of Women's and Children's Health, Karolinska Institutet, Stockholm, Sweden; 3 Orthopaedic Section, Department of Clinical Science and Education, Södersjukhuset, Karolinska Institutet, Stockholm, Sweden

**Keywords:** Osteoarthritis, Knee Osteoarthritis, Chondrocytes

## Abstract

**Objectives:**

It has been suggested that the lysosomal recycling process called macro-autophagy plays a role in osteoarthritis development. We thus decided to genetically ablate the autophagy-indispensable Atg5 gene specifically in chondrocytes and analyse the development of osteoarthritis upon aging and in a post-traumatic model.

**Methods:**

Mice lacking the Atg5 gene in their chondrocytes (Atg5cKO) were generated by crossing Atg5-floxed mice with transgenic mice that expressed cre recombinase driven by the collagen type 2 promoter. Animals were analysed at the age of 2, 6 and 12 months for age-related osteoarthritis or underwent mini-open partial medial meniscectomy at 2 months of age and were analysed 1 or 2 months after surgery. We evaluated osteoarthritis using the Osteoarthritis Research Society International (OARSI) scoring on safranin-O-stained samples. Cell death was evaluated by terminal deoxy-nucleotidyl-transferase-mediated deoxy-UTP nick end labelling (TUNEL) and by immunostaining of cleaved caspases.

**Results:**

We observed the development of osteoarthritis in Atg5cKO mice with aging including fibrillation and loss of proteoglycans, which was particularly severe in males. The ablation of Atg5 was associated with an increased cell death as assessed by TUNEL, cleaved caspase 3 and cleaved caspase 9. Surprisingly, no difference in the development of post-traumatic osteoarthritis was observed between Atg5cKO and control mice.

**Conclusions:**

Autophagy protects from age-related osteoarthritis by facilitating chondrocyte survival.

## Introduction

Macro-autophagy (thereafter referred as autophagy), which involves catabolic degradation of damaged organelles and long-lived protein complexes, promotes cell survival during nutritional depletion.^1^ This process involves phagophore formation with a double membrane, conjugation of Atg5 and Atg12 proteins, engulfment of cytosolic components and fusion of autophagosome with lysosome, where the contents are degraded and released into the cytosol. Conjugation of Atg5 and Atg12 is indispensable for autophagy.^2^ Ablation of Atg5 impairs autophagy and can diminish cell survival capacity.^3^ It was suggested that autophagy plays a role in the onset of osteoarthritis (OA).^4^ ^5^ In healthy human and murine cartilage, autophagy-related proteins unc-51 like autophagy activating kinase 1 (ULK1), Beclin1 and microtubule-associated protein 1A/1B-light chain 3 (LC3) are highly expressed in all the layers of articular cartilage,^4^ ^6^ but their expression decreases during OA.^4^ ^7^ On the other hand, in a rat model of surgically induced OA, the level of LC3-II is increased in the articular cartilage, indicating an increased autophagy.^10^ Mechanistic target of rapamycin complex 1 (mTORC1) intracellular signalling pathway is a master regulator of autophagy and activates autophagy when blocked.^8^ Inhibition of mTORC1 with either rapamycin or by targeted deletion of mTOR gene protects from surgery-induced OA in mice.^7^ ^9^

Thus, autophagy is clearly modulated during OA development, suggesting its role in this process. However, the direct evidences are still missing. Here, we explored OA development in mice without autophagy in their chondrocytes.

## Materials and methods

See more details in the online supplementary methods.

### Animal studies

Atg5cKO mice were generated by crossing Atg5-floxed mice with transgenic mice that expresses cre recombinase driven by collagen type 2 promoter (Col2-Cre mice). Mice of mixed background were used for the analysis.

### Statistical analysis

For all the experiments, each animal was considered as one observation. All staining were repeated five to ten times and averaged for every animal. All histological analysis was performed by ‘a blinded’ observer. Data are represented as mean values±SEM. Statistical analysis was done by unpaired Student's t test.

## Results

### Atg5 recombination in articular cartilage

**Figure 1:**
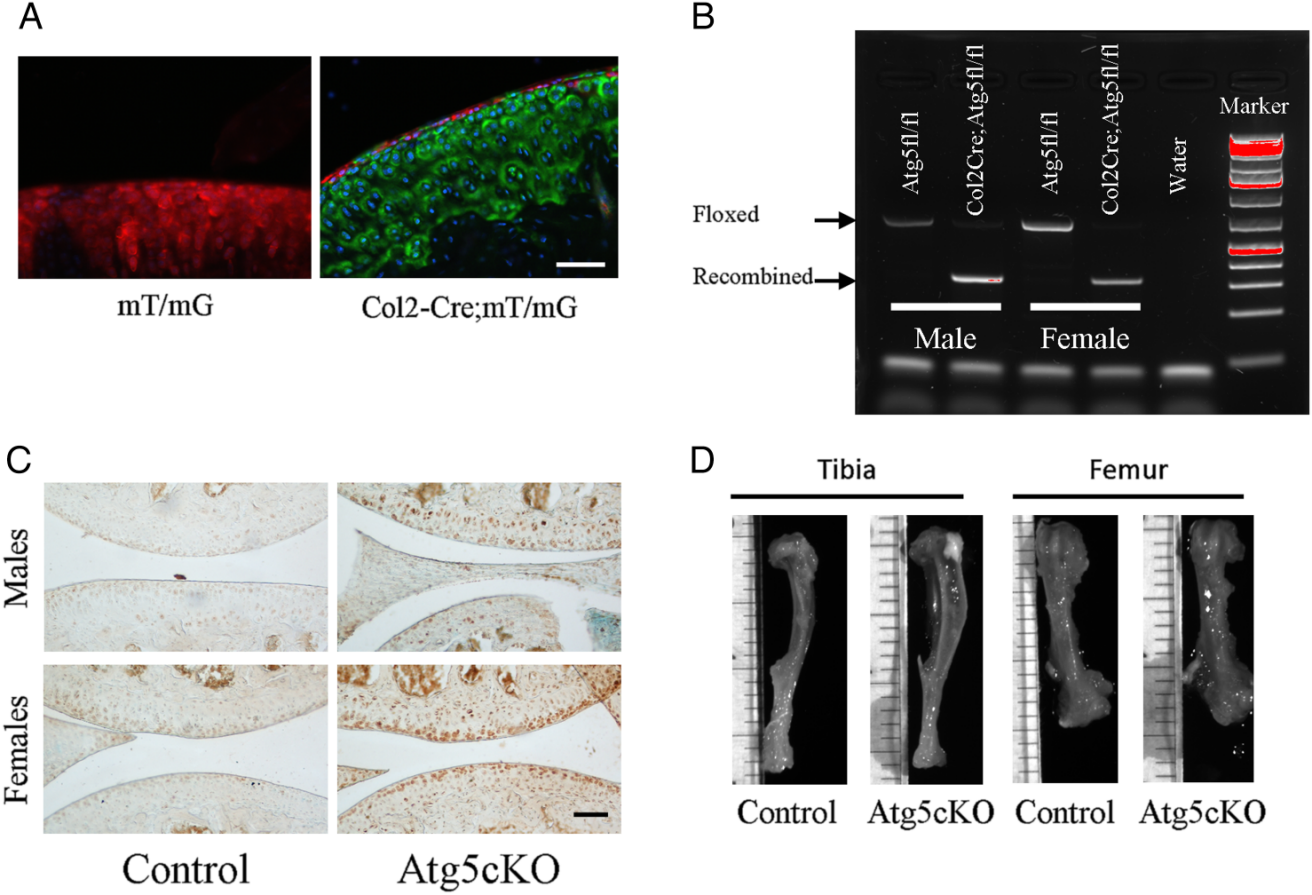
Efficiency of autophagy ablation in chondrocytes. (A) Representative photograph of the articular cartilage of mT/mG and mT/mG-Col2Cre mice. Upon Cre recombination, the fluorescence switches from red to green. (B) Gel showing the recombination of the Atg5 gene in the articular cartilage of Atg5cKO animals. (C) Immunostaining of P62 in the joint of control and Atg5cKO mice at 2 months of age. Bars=100 µm. (D) Representative pictures of 6-month-old bones of control and Atg5cKO animals.

Atg5cKO (Col2-Cre;Atg5fl/fl) mice appeared normal (figure 1D) with mild growth retardation associated with elevated death of epiphyseal chondrocytes.^11^ First, we verified the activity of Cre recombinase in articular chondrocytes by crossing Col2-Cre mice with mT/mG reporter mouse strain. Nearly 100% recombination was observed in the whole articular cartilage except the superficial zone (figure 1A). Next, we extracted DNA from the articular cartilage of control (Atg5fl/fl) and Atg5cKO mice and found recombination of Atg5 gene only in the Atg5cKO cartilage (figure 1B). P62 is a protein that accumulates upon autophagy inhibition and is used as a readout for autophagic activity.^12^ Accumulation of p62 was detected in the articular cartilage of Atg5cKO males and females, but not in control (figure 1C), confirming abrogated autophagic flux. At 6 months of age, Atg5 protein was detected by western blot only in the articular cartilage of the control (see online supplementary figure S1A). Accumulation of p62 was still detected in the articular cartilage of Atg5cKO mice at 6 months of age as compared with control, but less abundant than at 2 months of age (see online supplementary figure S1C).

### Age-related development of OA in Atg5cKO mice

**Figure 2:**
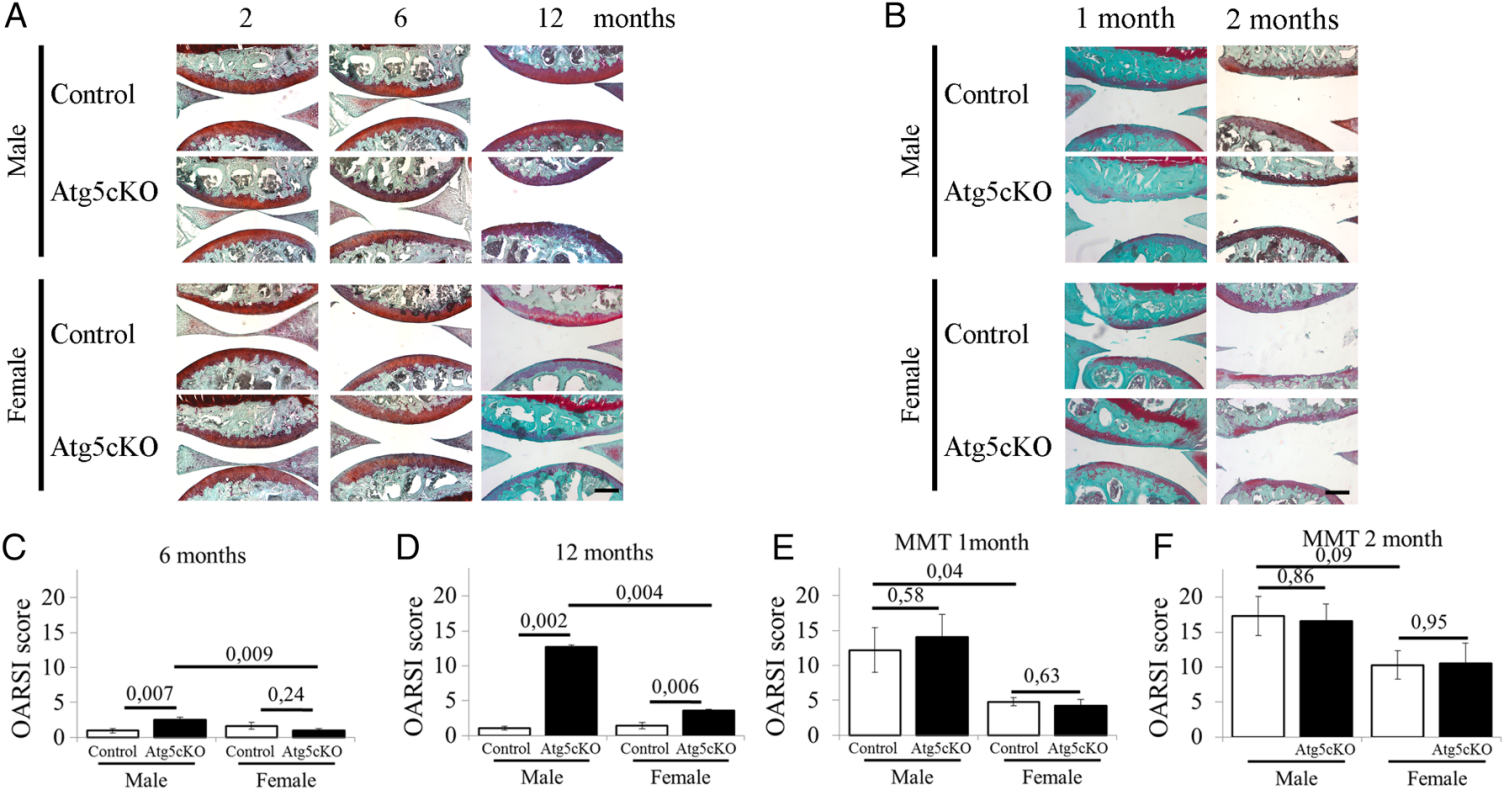
Histological scoring of control and Atg5cKO joints. (A) Representative photographs of the joints of male and female mice at 2, 6 and 12 months of age. (B) Representative photographs of male and female mice 1 and 2 months after partial medial meniscectomy (MMT). Osteoarthritis Research Society International (OARSI) scoring of the joints of control and Atg5cKO male and female mice at 6 (C) and 12 (D) months of age as well as 1 (E) and 2 (F) months after surgery. Sections were stained with Safranin O/Fast green. Two-month-old: n=6; 6-month-old: males n=5, females n=10; 1-year-old males n=7, females n=10; MMT, 1 months: n=8, 2 months: n=5. Values represent mean±SEM. Bars=200 µm.

At 2 months of age, both control and Atg5cKO joints had no sign of joint abnormality in both genders (figure 2A). At the age of 6 months, the first signs of fibrillation and loss of proteoglycans in the superficial zone were observed in male Atg5cKO joints (see figure 2A, C and online supplementary figure S2A). At the same time Col2-Cre; Atg5fl/+males were used as an additional control and showed no signs of OA development (data not shown). No signs of OA development were observed in Atg5cKO females at 6 months of age (see figure 2A, C and online supplementary figure S2B). At 1 year of age, Atg5cKO males developed substantial OA as compared with control males with fibrillation, a dramatic loss of Safranin-O staining and up to total erosion in 29% of the analysed animals (see figure 2A, D and online supplementary figure S2C). This was associated with an increase in matrix metalloproteinase (MMP)13 levels in the articular cartilage (see online supplementary figure S4C). The first signs of cartilage degradation were observed in female Atg5cKO mice at this time point (figure 2A–D). There was no difference in Osteoarthritis Research Society International (OARSI) score between male and female of the control group (figure 2A, D), whereas Atg5cKO males had a higher OARSI score than Atg5cKO females (figure 2C, D). No changes were observed in the synovium, subchondral bone or formation of osteophyte (data not shown). OA develops predominantly on the medial side in humans^13^ although it might be different in mice.^14^ Accordingly, we analysed the lateral side of 1-year-old males, but did not find any differences between control and Atg5cKO (see online supplementary figure S3).

### Post-traumatic OA in Atg5cKO mice

We then studied the role of autophagy in a post-traumatic model of OA. In both genders, no differences were seen between the control and Atg5cKO animals 1 month after surgery or 2 months after surgery (see figure 2B, E, F and online supplementary figure S2E–H). Upon surgery, males developed a more severe OA compared with females in both control and Atg5cKO genotypes. No differences were observed in synovial membrane inflammation or thickening, subchondral bone sclerosis or in osteophyte formation between control and Atg5cKO animals after surgery (data not shown).

### Atg5 ablation leads to caspase-mediated chondrocytes death

**Figure 3:**
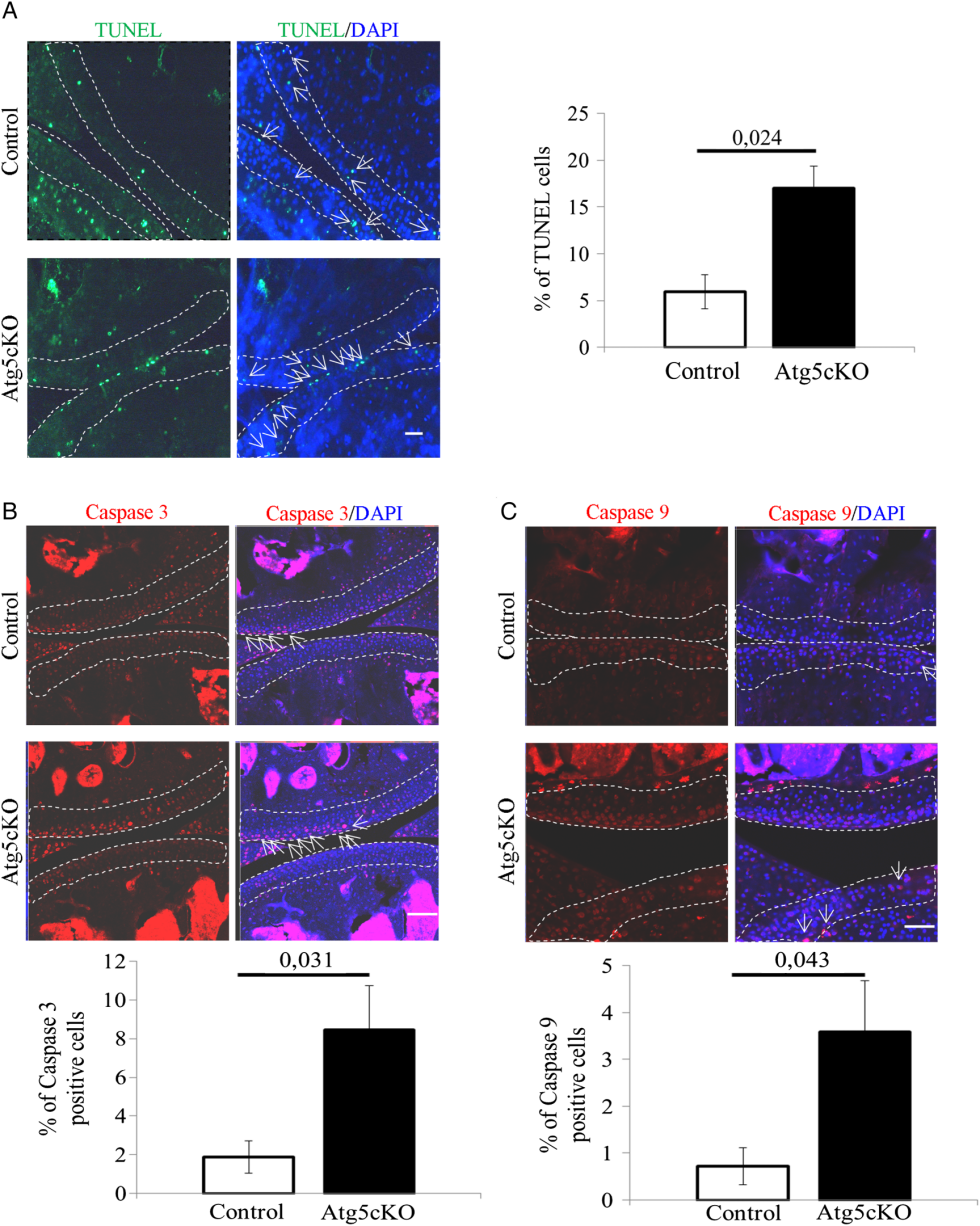
Cell death in the cartilage of control and Atg5cKO mice at 2 months of age. (A) Levels of terminal deoxy-nucleotidyl-transferase-mediated deoxy-UTP nick end labelling (TUNEL), (B) cleaved caspase 3 and (C) cleaved caspase 9 in control and Atg5cKO articular cartilage. n=6 (A) and n=5 (B and C). Values represent mean±SEM. Bars=100 µm. DAPI, 4′,6′-diamidino-2-phénylindole.

Autophagy is a process participating in cell survival.^3^ Accordingly, we analysed chondrocytes apoptosis in the articular cartilage of intact 2-months-old control and Atg5cKO mice. Atg5cKO mice had a higher cell death rate than control (figure 3A). We then assessed the level of cleaved caspase-3 and cleaved caspase-9 in chondrocytes. Atg5cKO had a higher number of chondrocytes positive for both cleaved caspase-3 (figure 3B) and cleaved caspase-9 (figure 3D). No differences in cell death were seen at 6 months of age (see online supplementary figure S1D). No difference in cellularity was found at 2 months of age (see online supplementary figure S4A). However, the cellularity decreased at 1 year compared to 2 months and the decrease was more pronounced in Atg5cKO animals (see online supplementary figure S4A, B).

## Discussion

Autophagy genes are regulated during aging and OA development^4^ suggesting that autophagy is involved in this process. Our data support this idea and provide the first direct evidence that autophagy prevents cartilage degeneration in physiological setting. Genetic ablation of Atg5 leads to the development of OA with age and is associated with caspase-dependent apoptosis. Autophagy is a cell survival mechanism, especially in a low nutrient environment.^3^ The articular cartilage is a low nutrient tissue,^15^ thus impaired viability of autophagy-deficient chondrocytes might be the underlying cause of OA in these mice. The underlying mechanism of cell death might include release of cytochrome *C* as we recently showed in the chondrogenic cell line RCJ 3.1C5.18.^11^

It is important to emphasise that cell death of articular chondrocytes is observed at 2 months of age, whereas the first signs of age-related OA become visible at 6 months. We believe that partial chondrocyte loss early in life facilitates OA development with age. Interestingly, despite the absence of Atg5, a partial restoration of p62 levels occurs at later time points, suggesting some compensatory mechanisms. We saw a similar compensation for Atg5 loss in epiphyseal chondrocytes.^11^ Theoretically, Atg5-independent autophagy^16^ or chaperone-mediated autophagy^17^ might compensate for Atg5 loss. This might also accounts for the absence of difference in chondrocyte death at later time points (ie, 6 months; online supplementary figure S1D). Thus, if anything, we rather underestimate the importance of autophagy for cartilage protection.

We did not observe an effect of autophagy ablation on the development of post-traumatic OA. In this model, cartilage degradation is very quick and might outweigh the mild degradation induced by ablation of autophagy. Inactivation of mTOR protects from post-traumatic OA presumably via activation of autophagy.^7^ ^9^ This is in line with our general observation that autophagy has a protective function in chondrocytes although experiments with mTOR must be interpreted carefully since mTOR has numerous additional actions such as regulation of protein synthesis, microtubule organisation, lipid synthesis, cytoskeleton organisation and mitochondrial metabolism.^8^

Post-traumatic OA is known to be more severe in males.^18^ However, it was surprising to see that Atg5cKO male mice are significantly more sensitive to age-related OA than female mice. It was recently reported that female mice are more sensitive to bone loss upon autophagy ablation in osteoblasts.^19^ On the other hand, ablation of Atg7 in osteocytes leads to more profound bone loss in males.^20^ Thus, there might be sex-related differences in response to autophagy ablation. The known protective effect of oestrogens on OA development^21^ might account for the difference between Atg5cKO males and females in our study. At present, the interaction between autophagic machinery and sex hormones remains an enigma.

In summary, our data show that autophagy protects articular cartilage and the abrogation of this process leads to the development of OA upon aging, especially in male mice. This effect is likely associated with a compromised viability of the chondrocytes.

## Supplementary Material

Web supplement
