# Supplementary material for: Targeted deletion of Atg5 in chondrocytes promotes age-related osteoarthritis
Source: Ann Rheum Dis. 2015 Oct 5;75(3):627–31. doi: 10.1136/annrheumdis-2015-207742 (PMC4789686; doi:10.1136/annrheumdis-2015-207742)

Supplementary Information

To the manuscript "Targeted deletion of Atg5 in chondrocytes promotes age-related osteoarthritis" by Thibault Bouderlique *et al.*

Supplementary methods

Ethical Statement

All animal studies were approved by the local ethical committee at Karolinska Institutet and all the animal studies were conducted according to the Swedish National Board for laboratory animals.

Reagents

Ethylene-diamine-tetra-acetic acid (EDTA), sucrose, Laemmli buffer 2x and DAPI were from Sigma-Aldrich Inc (St Louis, Missouri, USA). Phosphate buffers saline (PBS) and streptavidin-Alexa fluor 546 were from Life Technologies (Carlsbad, CA). Anti-p62 polyclonal guinea pig primary antibody was from Progen Bioteknik (Heidelberg, Germany). Antibodies to cleaved caspase-3 and 9, Beclin1 and Atg5 were from Cell Signalling Inc. (Danvers, MA). Antibody against MMP13 was from Santa-Cruz (Texas, USA). Secondary antibodies were from Jackson Laboratories (West Grove,PA). Vectastain ABC kit was from Vector Laboratories (Burlingame, CA). 3,3'-Diaminobenzidine (DAB) kit and horse serum were purchased from DAKO (Carpinteria, CA).

Animal Studies

Atg5cKO mice were generated by crossing Atg5-floxed[1] mice with transgenic mice that expresses cre recombinase driven by collagen type 2 promoter (Col2-Cre mice).[2] Mice of mixed background were used for the analysis. DNA recombination in articular cartilage was

assessed as previously described.[1] Knee joints were analysed at 2, 6 and 12 months. For surgically-induced osteoarthritis, Atg5cKO and control mice underwent a mini-open partial medial meniscectomy (MMT) at the age of 2 months and were analysed 1 and 2 months after surgery.[3] The right leg was operated while the left leg served as an internal control. mT/mG double fluorescent reporter mice,[4] which switch expression of tomato to GFP upon cre recombination, were crossed with Col2-Cre mice to generate Col2-Cre;mT/mG mice and analysed at two months of age. mT/mG joints were imaged using a confocal microscope (Zeiss LSM710).

Histology

Cryo-sections of intact knee joints were obtained and kept at -20ºC before analysis. Histomorphometric analysis was done on Safranin O stained sections according to the OARSI scoring.[5] Five sections of the medial joint separated approximately by 100µm distance were analysed for each animal. All analysis was made on encrypted slides.

Immunohistochemistry

Sections were blocked for 1 hour in 3% goat serum and primary antibody against p62 (1:1000) or MMP13 (1/200) or cleaved caspase-3 (1:50) or cleaved caspase-9 (1:50) were applied at 4°C overnight. For p62 and MMP13, visualization was made with corresponding secondary biotinylated antibody followed by incubation with avidin-biotin complex according to manufacturer’s instruction and DAB staining kit. For cleaved caspases, incubation with corresponding secondary HRP-conjugated antibodies followed by tyramide signal amplification (Perkin Elmer, Waltham, MA), streptavidin-Alexa fluor 546 visualization and DAPI counterstaining. Images were analysed by ImageJ software (NIH, Bethesda, MD).

Terminal deoxynucleotidyltransferase (TDT) - mediated deoxy-UTP nick end labelling (TUNEL) assay

For detection of apoptotic cells TUNEL labelling was performed according to the manufacturer's instructions (Fluorescein *In situ* cell death detection kit; Roche Inc, Stockholm, Sweden). Three sections were analysed for each joint.

Cell density assessment

Sections were thawed in PBS and stained with DAPI. After imaging, cells were counted and the area measured using ImageJ. Results are reported as number of cells/sqµm.

Western blot

Legs were dissected from 6 months old males and placed in ice-cold PBS before collection of the cartilage. Before removing the articular cartilage, the bones were imaged using a Chemidoc MP (BioRad). The articular cartilage was carefully separated from the surrounding bone and lysed directly in 2x Laemmli buffer, homogenized, ultra-sonicated and boiled. Samples were then submitted to an electrophoresis on SDS-polyacrylamide gel. Proteins were transferred to PVDF membranes and blocked for 1 hour with non-fat 5% milk. The membrane was incubated with primary antibodies overnight. Visualization was made by incubation with corresponding Horse Radish Peroxidase-labelled secondary antibodies followed and imaged using a Chemidoc MP (BioRad).

Supplementary bibliography

1 Hara T, Nakamura K, Matsui M, *et al.* Suppression of basal autophagy in neural cells causes neurodegenerative disease in mice. *Nature* 2006;**441**:885–9. doi:10.1038/nature04724

2 Kobayashi T, Chung U-I, Schipani E, *et al.* PTHrP and Indian hedgehog control differentiation of growth plate chondrocytes at multiple steps. *Development* 2002;**129**:2977–86.http://www.ncbi.nlm.nih.gov/pubmed/12050144 (accessed 15 Mar2015).

3 Knights CB, Gentry C, Bevan S. Partial medial meniscectomy produces osteoarthritis pain-related behaviour in female C57BL/6 mice. *Pain* 2012;**153**:281–92. doi:10.1016/j.pain.2011.09.007

4 Muzumdar MD, Tasic B, Miyamichi K, *et al.* A global double-fluorescent Cre reporter mouse. *Genesis* 2007;**45**:593–605. doi:10.1002/dvg.20335

5 Pritzker KPH, Gay S, Jimenez S a, *et al.* Osteoarthritis cartilage histopathology: grading and staging. *Osteoarthr Cartil OARS Osteoarthr Res Soc* 2006;**14**:13–29. doi:10.1016/j.joca.2005.07.014

Supplementary Figures:

Figure S1: Levels of autophagy and apoptosis at 6 months of age in control and Atg5cKO mice. (**A**) Western blot showing the absence of Atg5 in the articular cartilage of Atg5cKO animals at 6 months of age. (**B**) Quantification of TUNEL-positive cells in the articular cartilage at 6 months of age. (**C**) Immunostaining of P62 in the joint of control and Atg5cKO mice at 6 months of age. Bars = 100 µm.

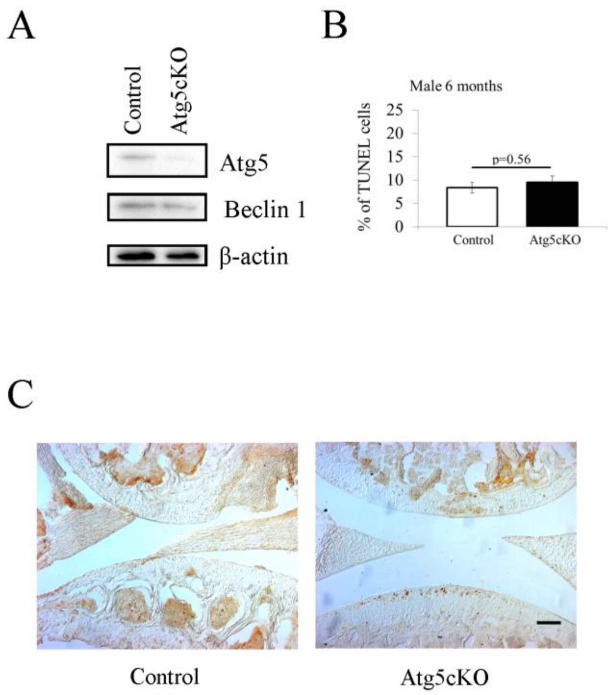

Figure S2: Histologic scoring of control and Atg5cKO tibial and femoral articular cartilage. OARSI scoring of the joints of control and Atg5cKO male (**A, C, E, G**) and female (**B, D, F, H**) mice at 6 (**A, B**) and 12 months (**C, D**) of age as well as one (**E, F**) and two (**G, H**) months after surgery. 6months of age: males n=5, females n=10, 1year of age males n=7, females n=10, MMT, 1 months: n=8, 2 months n=5.Values represent mean ± SEM, p-values are displayed on the graphics. Bars = 200 µm.

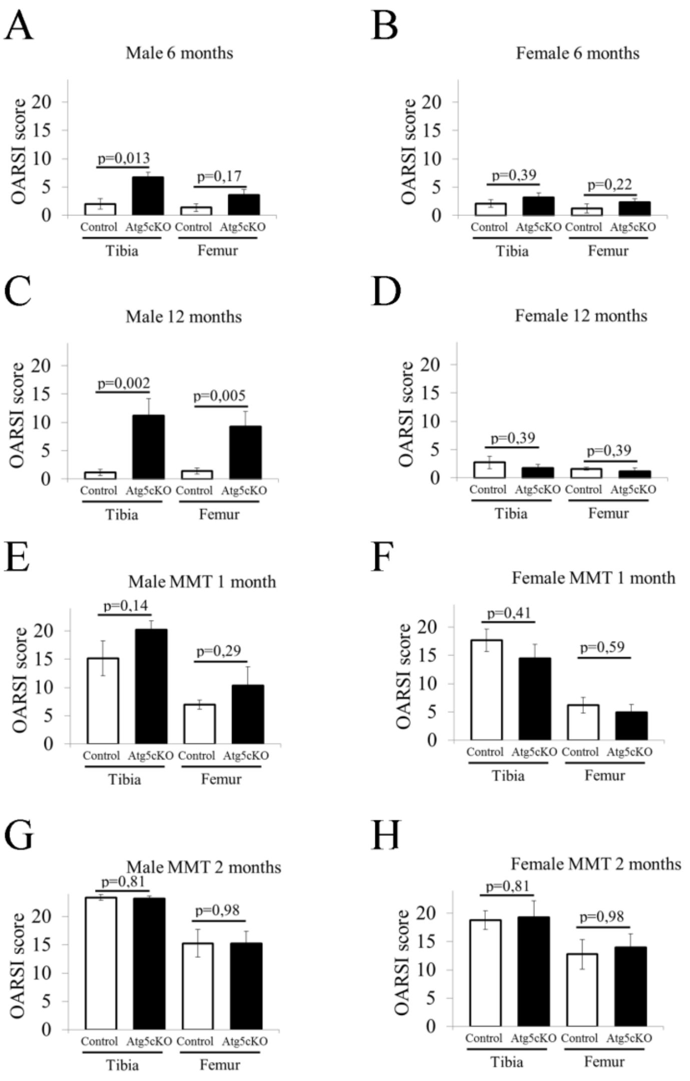

Figure S3: Histologic scoring of control and Atg5cKO of the lateral compartment of tibial and femoral articular cartilage at 12 months of age.

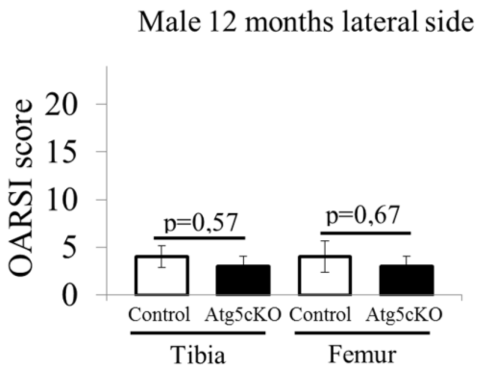

Figure S4: Cell density and MMP13 expression in 12-months-old males. Cell density in the articular cartilage at 2 (**A**) and 12 (**B**) months of age. (**C**) MMP13 immunostaining of the articular cartilage at 12 months of age.

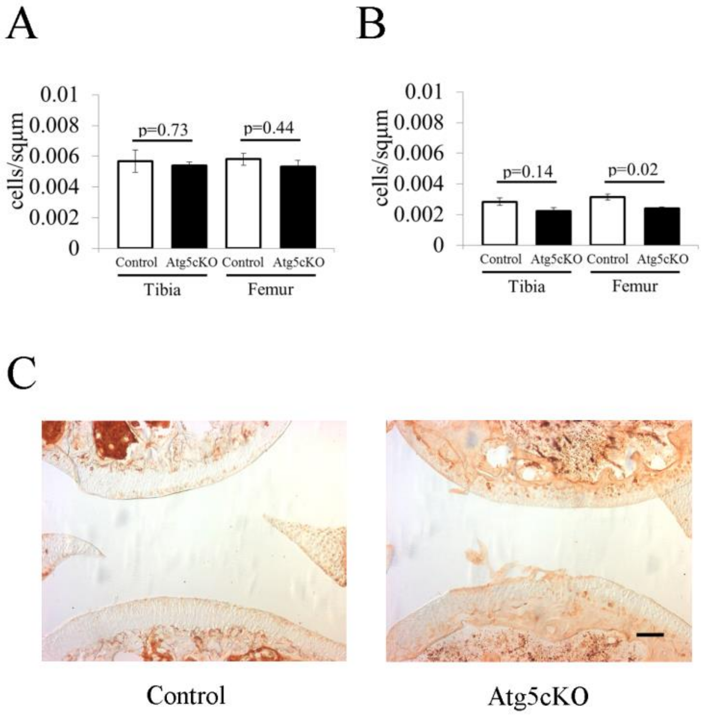

Supplement: Web supplement [file annrheumdis-2015-207742-s1.pdf]
